# Evaluation of Cross-Linked Polyamide 6 Micro-Indentation Properties: TAIC Concentration and Electron Radiation Intensity

**DOI:** 10.3390/ma16062391

**Published:** 2023-03-16

**Authors:** Martin Ovsik, Michal Stanek, Martin Bednarik

**Affiliations:** Faculty of Technology, Tomas Bata University in Zlin, Vavreckova 5669, 760 01 Zlín, Czech Republic

**Keywords:** polyamide 6, cross-linking, electron radiation, micro-indentation test, indentation hardness, structural properties

## Abstract

Nowadays, technical practice puts emphasis on improving selected material properties of polymers which could lead to new applications. Material properties can be modified in numerous ways, among which is radiation treatment. This study looks into the influence of beta radiation on several properties of polyamide 6, e.g., indentation hardness, modulus and creep. Main changeable parameters were the concentration of triallyl isocyanurate (TAIC), which promotes cross-linking, and intensity of radiation. The concentration was in the range from 2 to 6 wt.%, while the radiation dose was 0, 66, 99 and 132 kGy. The treated materials were measured for indentation hardness, modulus and creep. Degree of cross-linking was verified by thermo-mechanical analysis (TMA), while degradation processes was investigated by Fourier-transform infrared spectroscopy (FTIR). The results indicate that electron radiation positively affects the tested material properties. The best results were seen in polyamide with 6 wt.% of TAIC, which demonstrated a 38% improvement in mechanical properties after exposure to 132 kGy. This improvement in properties affects the final parts and their application (e.g., in the automotive industry—engine parts; in electrical engineering—insulation of wires and cables; and in industry—pipes for underfloor heating, etc.).

## 1. Introduction

This study is focused on PA 6 modified with differing concentration of cross-linking agent (CLA) irradiated with three varying doses. Industry puts emphasis on material improvement, one of the methods of which is irradiation. The literature sources laid out in this research focus on the characterisation of radiation influence on mechanical properties of polyamide 6. On the other hand, these articles lack sufficiently complex information regarding this topic. Some correlation between ionising beta radiation, polymer morphology and changes in mechanical properties are hard to find, even though this information is necessary for industrial application of irradiated polyamides with CLA.

Overall, resistance of polyamides to ionising radiation is intermediate. It is lower in type –CONH–(CH_2_)n– polyamides, which have a higher number of methyl groups within peptide bonds. As a result of ionising radiation, both cross-linking and degradation of chains commences. If the cross-linking prevails over degradation, then the irradiated material can be improved [1].

Construction of 3D networks is not too strong. The presence of oxygen leads to scission of polyamide chains and the creation of peroxides. Kaindle and Graul show [2], that free radicals are produced during irradiation, especially in Structures (1) and (2).

(1) $$-\mathrm{CONH}-C^{*}H-{\mathrm{CH}}_{2}-$$

(2) $$-C^{*}O-\mathrm{NH}-{\mathrm{CH}}_{2}-$$

Radicals are also produced in C=N and C=C double bonds. Rexer stated [3] that the dose that leads to the creation of gel in the presence of oxygen is 350 kGy. Irradiation in vacuum leads to structure changes that result in improved mechanical and thermo-mechanical properties. The addition of poly functional monomers, such as TAIC, can cause the polyamides to cross-link after exposure to relatively low doses of radiation, even in the presence of oxygen. Finding the way in which these doses influence selected mechanical properties is the topic of this study. Hydrogen on carbon neighbouring with nitrogen in amide group is taken out and water, carbon monoxide and dioxide and methane are created. Three allyl groups of TAIC can react due to created macro-radicals in polyamide, creating a 3D network [3].

Aside from cross-linking, the irradiation of polyamides in the presence of oxygen (typical interceptor of radicals) also leads to fragmentation, disproportion and oxidation, resulting in scission of chains in the microscopic world and embrittlement in the macroscopic world. An initial study of the oxidation behaviour of polyamides was conducted by Sharkley, Mochel, Levantovskaya, Lock and Sagar. The mechanism was based on primary engagement of methyl groups neighbouring with –NH-groups. The radicals created on these groups subsequently react with oxygen, which leads to the creation of more radicals, which can either isomerise or undergo other reactions, including both the cross-linking and scission of polymer chains. All of these reactions eventually lead to the creation of final carbonyl or carboxyl groups [4,5]. The scission of polymer chains leads to a reduction in the number of free amino groups [6].

Radiation cross-linking of polyamides has been researched in numerous studies [6,7,8,9,10], but these studies do not contain sufficient amounts of complex information, and it is hard to obtain the required interactions among electron radiation and morphological changes from them.

The authors of this study examined the influence of electron radiation on the morphological, thermal and mechanical properties of polyamide 6. The material was exposed to radiation of up to 150 kGy. Properties were improved in the specimen irradiated by 75 kGy, but more than that led to degradation [11]. Shin et al. investigated PA6 irradiated by beta radiation with maximum intensity 200 kGy. The results showed that mechanical properties improved, and especially elastic modulus rose by 47% in comparison with unaltered PA6 [12]. These findings are consistent with the results of this study.

Shin et al. [13] studied the effect of beta radiation on the morphological, rheological and mechanical properties of polymer blend composed of PA 6 and PP. It was found that electron radiation improved properties, although they were not measured in the surface layer.

Bradler et al. [14] tested commonly accessible virgin and filled PAs with 30 and 35 wt.% glass fibres and 5 wt.% TAIC. These samples were irradiated with 50, 100, 150 and 200 kGy. Contrary to the aforementioned study, in this case, the variation in TAIC concentration was smaller, while the radiation doses varied more widely.

Studies [15,16] have shown that irradiation can lead to improved mechanical properties, and that the addition of cross-linking agent, e.g., TAIC, enhances this effect. These studies confirm the findings presented in this work.

Other authors [17,18,19,20] have investigated the effect of beta radiation on the mechanical and thermal properties of the observed polymer types. It was found that finding the optimal dose of radiation always leads to improved material properties. Numerous polymers are investigated in this study. This study focuses on varying concentrations of CLA in PA 6, in order to obtain a more detailed description of this problem.

The measured results construct a basis upon which a more detailed study of this problem can be built. Finding deeper correlations of various dependencies requires a higher degree of repeatability during measurements, which is more demanding of time and finances.

Overall, this study deals with the influence of varying concentrations of cross-linking agents (2, 4 and 6%) on the micro-mechanical properties of irradiated polyamide 6. Individual samples were irradiated by electron radiation with differing intensities (66, 99 and 132 kGy). The main goal of this work is to find the optimal concentration of CLA and the optimal radiation dose at which to improve micro-mechanical properties. Suitable processing conditions can lead to more economical processes, thus generating better profit. The degree of irradiation was evaluated by thermo-mechanical analysis (TMA), while the degree of polyamide degradation was investigated by Fourier-transform infrared spectroscopy (FTIR).

## 2. Materials and Methods

The tested polyamide 6 was injection moulded with three different concentrations of CLA (0, 2, 4 and 6 wt.%). These samples were then irradiated by an electron beam with differing intensities (66, 99 and 132 kGy). Following manufacturing, the samples were measured with respect to their mechanical properties, i.e., indentation hardness, modulus and creep. Degree of irradiation was evaluated by thermo-mechanical analysis (TMA), while the occurrence of the degradation processes taking place was proved by means of Fourier-transform infrared spectroscopy (FTIR). Each sample combination was measured ten times, and these results were subsequently statistically evaluated by arithmetic mean and standard deviation. Table 1 presents the designation of individual samples.

### 2.1. Material

Polyamide 6 with commercial designation PA 6 Frianyl B63 VN made by Frisset (Utzenfeld, Germany) was chosen as the experimental material. This material has a broad range of application in industrial practice, and is often used for complex parts. Polyamide 6 is a semi-crystalline polymer with high strength. This material was modified through the addition of TAIC in the following concentrations: 2, 4 and 6 wt.%.

### 2.2. Sample Preparation

Test samples were manufactured by injection moulding in an Arburg Allrounder 470 H injection machine, which was made by Arburg (Losburg, Germany). Test samples were prepared in accordance with the ČSN EN ISO 179-1 standard in the shape of a rectangular block with dimensions 4 × 80 × 10 mm. The injection moulding parameters were set according to the manufacturer’s recommendations, as provided on the material sheet. This information can also be found in Table 2.

Polyamide 6 is characterised by its tendency to absorb water, which means it is necessary to dry the material before injection moulding. The drying process was performed in a Thermolift 100-2 dryer made by Arburg (Losburg, Germany). The parameters of the drying process were as follows: drying temperature 120 °C and time of drying 4 h. Material was dried and injected in the form of granules, which were provided by the manufacturer. Transport of the material from the dryer to the injection machine was carried out using a pneumatic transporter in a manner such that contact with additional humidity was prevented.

### 2.3. Sample Irradiation

Prepared samples were irradiated in cooperation with BGS Beta Gamma Service GmbH & Co., KG company (Saal an der Donau, Germany). Radiation was applied in a high-voltage accelerator of the Rhodotron type with a maximal energy output of 10 MeV. Radiation doses were set within the range commonly found in practice (66, 99 and 132 kGy), and irradiation was performed at ambient temperature in a normal atmosphere. Polyamide 6 was enhanced by TAIC in 2, 4 and 6 wt.%. TAIC enables chemical bonding of free radicals to applied CLA, leading to a certain degree of cross-linking.

### 2.4. Micro-Indentation Properties

**Figure 1:**
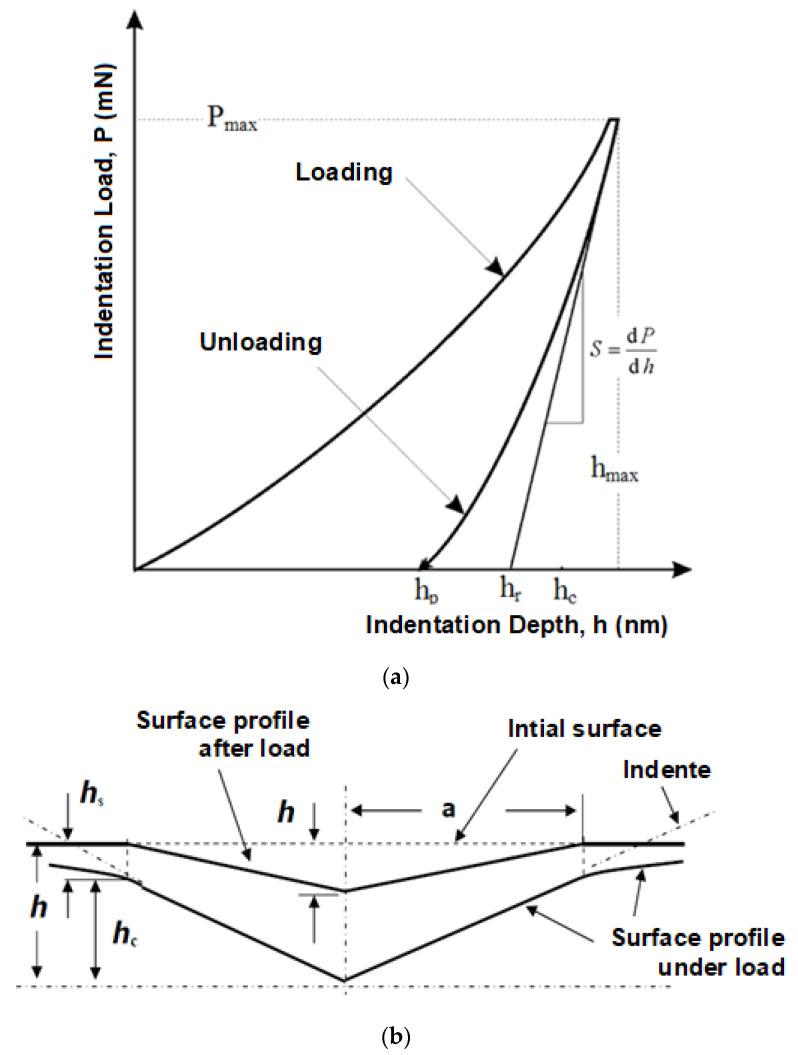
Principle of indentation: (**a**) load vs. indentation depth; (**b**) schematic representation of the indentation processes showing the decreasing of the indentation depth during loading (according to Oliver and Pharr).

Micro-indentation properties were measured using an MCT^2^ instrumented hardness meter provided by CSM (Graz, Austria). Parameters for the measurement were set as follows: applied load 1 N, loading and de-loading speed 2 N/min, maximum load duration 90 s. A Vickers four-sided pyramid with tip angle 136° was chosen as the indentation tip. This measurement was realised in accordance with the ČSN EN ISO 14577-1 standard, and indentation hardness, modulus and creep were evaluated based on this standard. All obtained parameters were evaluated using the method of Oliver and Pharr (Figure 1). The derivation of the measured mechanical properties can be seen below.

Indentation hardness *H_IT_* measures the resistance to permanent deformation or damage, where *F_max_* is the maximum force and *A_p_* is the projected contact area (theoretical or calibrated) [20,21,22].

(3) $$H_{IT}=\frac{F_{max}}{A_{p}}$$

(4) $$A_{p}=23.96\cdot h_{c}^{2}$$

As described in the ISO 14577 standard, the reduced modulus, *E_r_*, is used to account for the fact that the elastic displacements occur in both the indenter and the sample. The instrumented elastic modulus in the test material, *E_IT_*, can be calculated from *E_r_* using the following formula [20,21,22]:

(5) $$E_{IT}=E^{*}\cdot (1-{\nu_{s}}^{2})$$

(6) $$E^{*}=\frac{1}{\frac{1}{E_{r}}-\frac{1-\nu_{i}^{2}}{E_{i}}}$$

(7) $$E_{r}=\frac{\sqrt{\pi }}{2\cdot C\sqrt{A_{p}}}$$

where *ν_s_* is the Poisson’s ratio of the sample (polymer 0.3 to 0.4), and *E_i_* and *ν_i_* are the elastic modulus (diamond 1141 GPa) and Poisson’s ratio (0.07), respectively, of the indenter.

**Figure 2:**
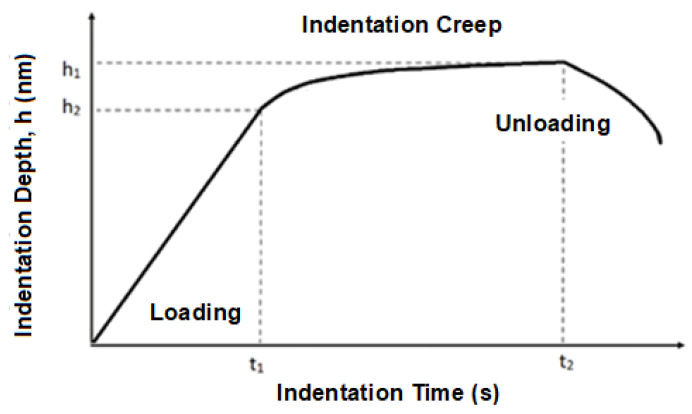
Indentation creep.

The instrumented hardness test allows the measurement of changes in imprint depth under constant load. The results can be used to calculate relative depth of imprint, i.e., creep. Indentation creep can be calculated using the following equation [20,22]:

(8) $$C_{IT}=\frac{h_{2}-h_{1}}{h_{1}}100$$

where *h*_1_ is the depth of imprint in time *t*_1_ after the testing load is reached, and *h*_2_ is the depth of imprint in time *t*_2_ that is reached at maximal testing load *P*_max_ (Figure 2) [22].

### 2.5. Thermo-Mechanical Analysis (TMA)

**Figure 3:**
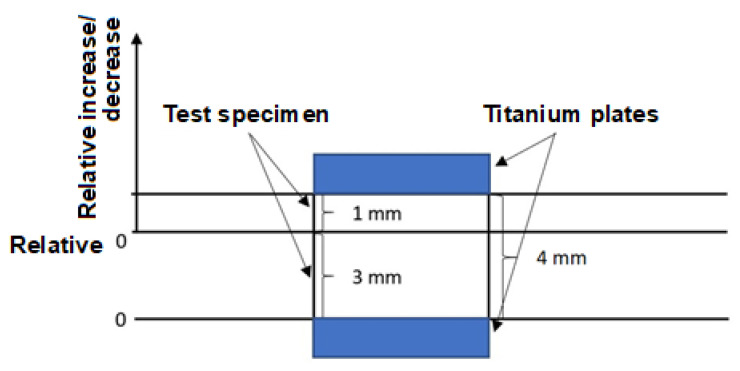
TMA measurement principle.

Measurement of the degree of polyamide 6 cross-linking was performed by thermo-mechanical analysis using a DMA made by Mettler Toledo (Langacher, Greifensee, Switzerland). The measurement was performed on three test samples with dimensions 10 × 4 × 10 mm for all TAIC concentrations and radiation doses. The process parameters are shown in Table 3 and Figure 3. Deformation calculation was carried out according to Equation (9).

(9) $$\frac{\mathrm{deformation}\;\mathrm{of}\;\mathrm{the}\;\mathrm{sample}\;\left(µm\right)}{\mathrm{thickness}\;\mathrm{of}\;\mathrm{the}\;\mathrm{sample}\;\left(µm\right)}\times 100=\mathrm{deformation}\;\left(\%\right)$$

### 2.6. Fourier-Transform Infrared Spectroscopy (FTIR)

Measurement of FTIR spectra was carried out on an AVATAR 320 made by Nicolet (Watertown, MA, USA). The method used was ATR with ZnSe crystal (zinc selenide). The background was scanned before and after each sample, and subsequently deducted in order to obtain more accurate measurements. This was done on three test samples with each TAIC concentration and radiation dose.

## 3. Results

The goal of this study was to show the effect of CLA concentration and radiation intensity on the indentation hardness, modulus, and creep of polyamide 6. The main goal was to find the optimal concentration of CLA and radiation intensity to be able to achieve a significant improvement in the mechanical properties of the surface layer of a commonly used construction polymer (polyamide 6). Micro-hardness was measured as a part of the measurements of micro-mechanical properties. This test is sufficiently sensitive to catch even small changes in observed properties.

### 3.1. Micro-Indentation Properties

**Figure 4:**
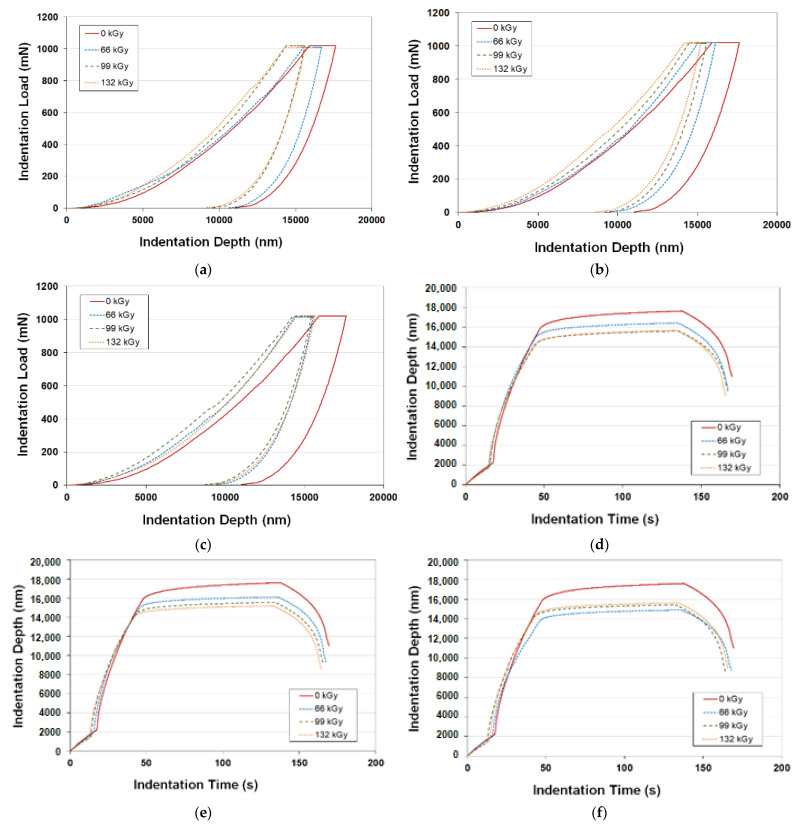
Indentation characteristics: (**a**) indentation force vs. depth of indentation—concentration of TAIC 2%; (**b**) indentation force vs. depth of indentation—concentration of TAIC 4%; (**c**) indentation force vs. depth of indentation—concentration of TAIC 6%; (**d**) depth of indentation vs. time of indentation—concentration of TAIC 2%; (**e**) depth of indentation vs. time of indentation—concentration of TAIC 4%; (**f**) depth of indentation vs. time of indentation—concentration of TAIC 6%.

The most important area of this research is the measurement of micro-mechanical properties, which was performed using contemporary technology, i.e., via the instrumented hardness test. This test provides a complex description of the mechanical behaviour of polymer materials in a non-destructive way. The material characteristics evaluated were indentation hardness, modulus and creep. These values can be obtained from the characteristics given in Figure 4.

Figure 4a–c present the indentation characteristics of indentation force as a function of depth of indentation for individual concentrations of CLA and radiation doses. This dependence provides initial information about polymer behaviour and represents a basis for the calculation of indentation hardness and modulus. It can be stated that more leftwards dependencies signify improved mechanical properties, because the indentation reached a lower depth.

Figure 4a–c show the indentation characteristics of indentation depth as a function of indentation time. When the maximum indentation force (1 N) is reached, it stays the same for a predetermined duration, during which the indentation depth increases. The indentation depth is determined based on these changes in indentation depth. The angle of the curve representing this process is used to determine the creep behaviour of the individual materials.

Hardness is one of the most important parameters for describing the mechanical behaviour of the tested surface layer of polyamide 6. With increasing hardness, the product can be used in more demanding applications, as it will present higher resistance against scratching and tearing.

**Figure 5:**
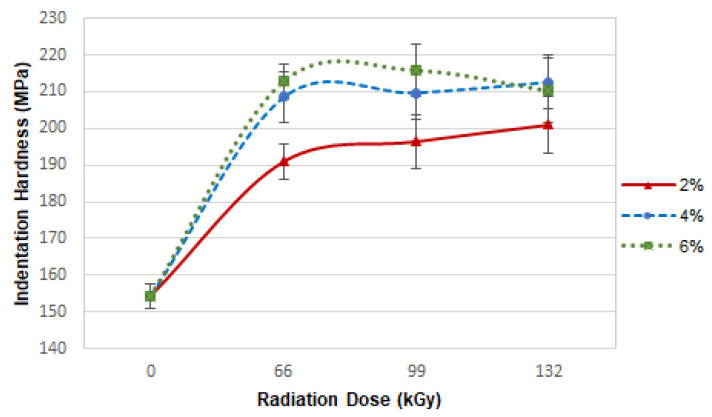
Dependence of indentation hardness on radiation intensity and TAIC concentration.

The indentation hardness results indicate that both the concentration of CLA TAIC and the radiation intensity have a significant effect on indentation hardness (Figure 5). The indentation hardness of sample A was 154 MPa. The indentation hardness in sample B increased with increasing radiation intensity, which was capped at 132 kGy. The most significant increase was found in the sample irradiated with 66 kGy (190 MPa). In subsequent samples, further irradiation led to smaller, although still significant, increases. Indentation hardness was 196 MPa in the sample irradiated by 99 kGy and 200 MPa in the sample irradiated by 132 kGy. There was a 30% difference between sample A and sample B irradiated by 132 kGy. The greatest increase in indentation hardness for sample C was found in the sample irradiated by 66 kGy (209 MPa). Samples irradiated at greater intensity displayed quite similar indentation hardness values. This value was 36% higher for the sample irradiated by 66 kGy than it was for sample A. Sample D was measured as possessing an indentation hardness of 213 MPa following irradiation by 66 kGy. In comparison with the base material, this was a 38% increase. Higher intensities led to decrease in indentation hardness values, which was due to the degradation processes occurring with high radiation doses.

The indentation hardness results indicate that the optimal radiation for polyamide 6 improvement was 66 kGy, while the optimal concentration of TAIC was 4 or 6 wt.%. Higher radiation intensity made indentation hardness more likely to decrease to increase.

A further critical parameter for characterising the mechanical behaviour of materials is elastic modulus. Great material resistance can be achieved by improving the elastic modulus of the tested polyamide 6.

**Figure 6:**
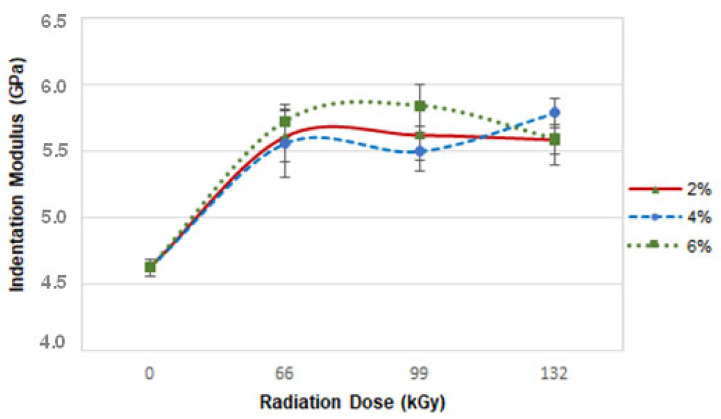
Dependence of indentation modulus on radiation intensity and TAIC concentration.

The results of elastic modulus indicate that both CLA concentration and radiation intensity have a significant effect on indentation modulus (Figure 6). The indentation modulus of sample B irradiated by 66 kGy was 5.5 GPa. Similar tendencies were found in samples irradiated by 99 and 132 kGy. The difference between the virgin (0 kGy) polyamide 6 (4.6 GPa) and the irradiated one (66 kGy) was 20%. The highest indentation modulus value obtained by samples C (5.8 GPa) was found for the sample irradiated by 132 kGy. The difference between the base material and sample C irradiated by 132 kGy was 26%. The best improvement in indentation modulus (5.8 GPa) was found in sample D irradiated by 99 kGy. In comparison with the virgin material, this represented a 26% increase. Indentation modulus decreased at 132 kGy, mostly likely as a result of degradation processes occurring due to the high intensity of the radiation.

Indentation creep is an important parameter for characterising material under long-term load (use). On the basis of indentation creep, it is possible to estimate how the product will behave in practice over a longer period of time.

**Figure 7:**
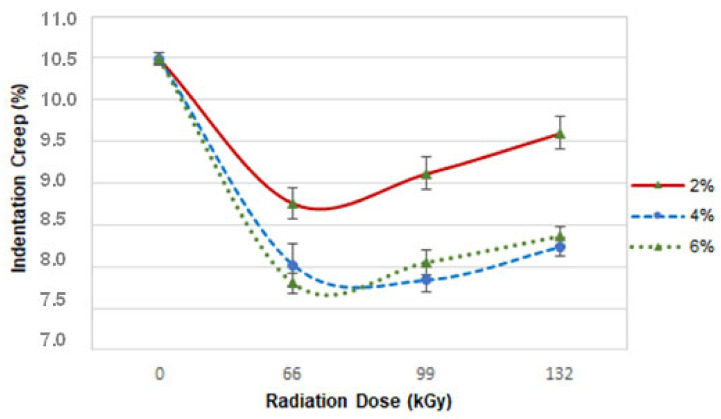
Dependence of indentation creep on radiation intensity and TAIC concentration.

Indentation creep tendency was similar for all radiation intensities (Figure 7). The base material had a creep of 10.5%. The most interesting development in indentation creep was measured in samples irradiated by 66 kGy. On the other hand, the indentation creep values became worse with increasing intensity. However, in the end, samples D demonstrated the best results, especially when irradiated with 66 kGy, following which the value of indentation creep was 7.7%. The difference between virgin material and that irradiated by 66 kGy was 36%.

This significant change in the behaviour of polyamide 6 was caused by the cross-linking of the polymer. The cross-linking of polymers is a chemical process, during which transverse bonds are created in the polymer structure. Infinite 3D structure, i.e., spatial network or gel, is produced due to these transverse bonds. Cross-linking induced by radiation is a result of the numerous free radical polymers moving around, especially in amorphous regions. During cross-linking, two opposite events occur simultaneously, i.e., cross-linking and degradation (scission of the main chain). The cross-linking of a polymer and its degree of cross-linking depend on many factors, including crystallinity, glass transition temperature, chemical structure of the polymer, etc.

### 3.2. Thermo-Mechanical Analysis (TMA)

**Figure 8:**
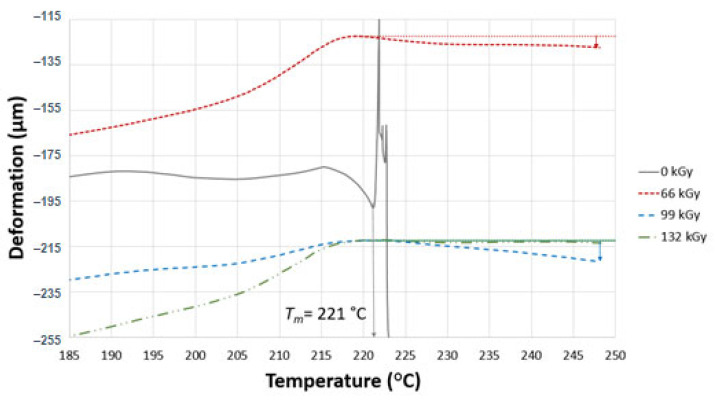
TMA of sample B.

**Figure 9:**
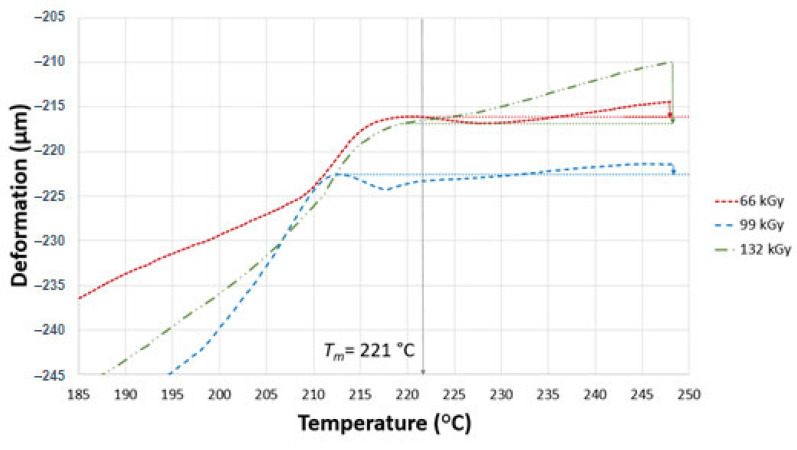
TMA of sample C.

**Figure 10:**
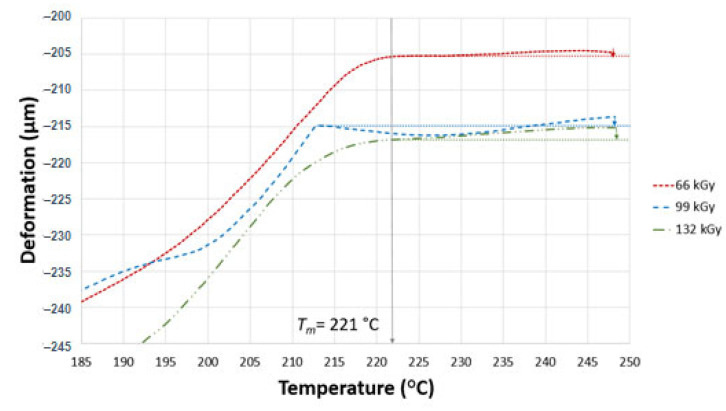
TMA of sample D.

TMA was performed to confirm the degree of irradiation of the test samples. In the case of TMA, two parameters were measured, i.e., thermal expansion and the flow of the non-cross-linked part. These two parameters influence one another, and are dependent on the currently dominating one. The curve can either go up due to thermal expansion or down due to thermal degradation. Similar results of deformations were found in all test samples, as can be seen in Figure 8, Figure 9 and Figure 10. The curves in the graphs were generated from the median values of three measurements. In order to provide better a picture of the measured trend, deformation was determined at different temperatures (230, 240 and 248 °C).

Figure 8 displays the deformation of samples A and B. As can be seen, the virgin sample (0 kGy) exhibited a melting temperature of 221 °C. On the other hand, the irradiated polyamide 6 showed different mechanical behaviour from the virgin material. The interrupted line represents a tangent that indicates where the deformations were taken from at all three temperatures (230, 240 and 248 °C). The deformation of the test sample started to rapidly increase when the temperature increased beyond 217 °C, and this phenomenon remained in effect until a temperature of 248 °C. The test sample exposed to 66 kGy showed improved mechanical behaviour with increasing temperature. Deformation was −0.09% at 230 °C, −0.1% at 240 °C and −0.12% at 248 °C.

The test sample irradiated by 99 kGy exhibited differences similar to those in the previous sample. The deformation was −0.06% at 230 °C, −0.15% at 240 °C and −0.23% at 248 °C. This slightly higher deformation in comparison with the sample irradiated by 66 kGy could be a result of insufficient cross-linking in the entire volume.

The highest radiation intensity (132 kGy) resulted in the lowest deformation of all of the test samples B. The measured deformation was −0.02% at 230 °C, −0.01 at 240 °C and −0.03% at 248 °C. On the basis of the mechanical behaviour angle, this radiation dose resulted in improved cross-linking compared to earlier test samples.

Figure 9 presents the course of TMA analysis for sample C. Deformation grew by −0.2% with the increase in temperature from 222 °C to 230 °C and after this temperature, thermal expansion began to dominate, as manifest by the curve growth. Deformation was 0.01 at 240 °C, while the thermal expansion was 0.04% at 248 °C.

Polyamide 6 irradiated by 99 kGy exhibited a slight decrease at 218 °C, which was probably caused by the incorrect clamping of the test sample between the titan discs. Higher temperatures enabled the domination of thermal expansion to be observed, which was 0% at 230 °C, 0.02% at 240 °C and 0.03% at 248 °C.

PA 6 exposed to 132 kGy exhibited the dominance of thermal expansion from a temperature of 220 °C. Thermal expansion was 0.05% at 230 °C, 0.12% at 240 °C and 0.17% at 248 °C. There was no deformation in this case. Value of thermal expansion as high as those measured makes it likely that the polyamide 6 was adequately cross-linked, and there was need to neither add more TAIC nor increase radiation intensity.

Figure 10 presents the thermo-mechanical analysis of sample D. This sample, irradiated by 66 kGy, exhibited the best balance of thermal expansion and flow of the non-cross-linked part. Thermal expansion was 0% at 230 °C, 0.02% at 240 °C and 0.01% at 248 °C. No significant deformation was observed, here.

The test sample irradiated by 99 kGy showed deformation from a temperature of 213 °C. This deformation prevailed until 227 °C, where thermal expansion started, as well. At 230 °C, deformation was still dominating, at −0.03%. At 240 °C, deformation was zero and thermal degradation was 0.01%. Thermal expansion at 248 °C was 0.03%.

PA 6 irradiated by the highest radiation dose showed thermal expansion of 0.01% at 230 °C, 0.04% at 240 °C and 0.04% at 248 °C.

The thermo-mechanical analysis results for sample C exposed to 132 kGy led to significant cross-linking. Samples D cross-linked after exposure to all radiation doses, and therefore they all demonstrated significant improvement in their mechanical property values.

### 3.3. Fourier-Transform Infrared Spectroscopy (FTIR)

**Figure 11:**
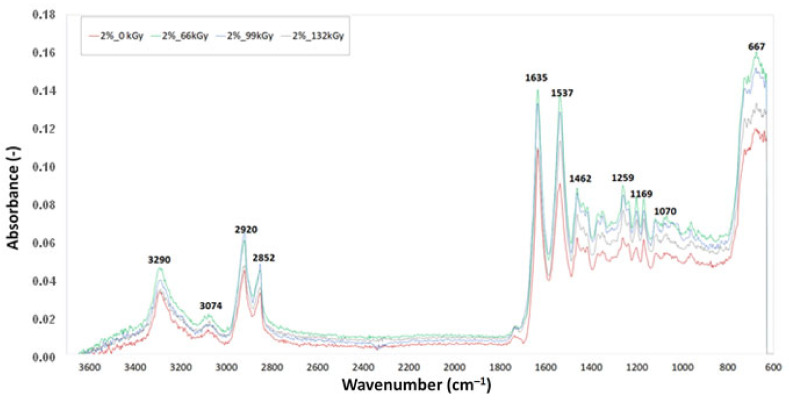
Fourier-transform infrared spectroscopy of sample B.

**Figure 12:**
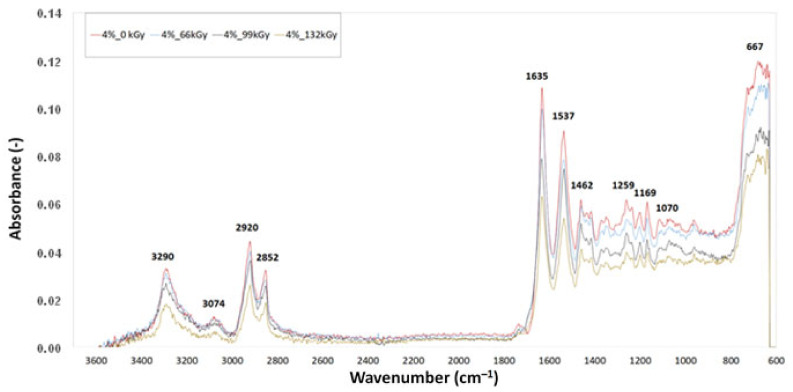
Fourier-transform infrared spectroscopy of sample C.

**Figure 13:**
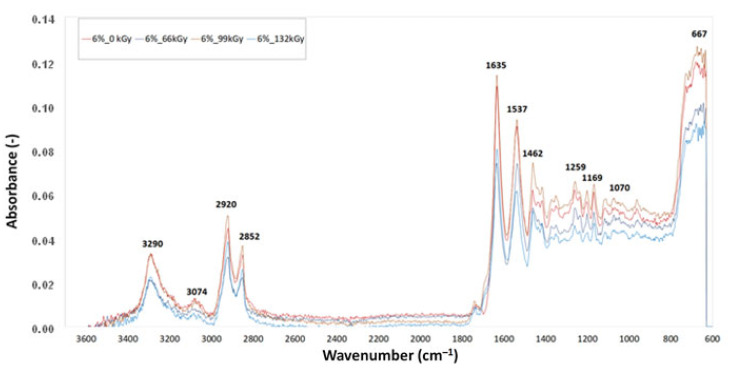
Fourier-transform infrared spectroscopy of sample D.

Figure 11, Figure 12 and Figure 13 display the FTIR analysis of the test samples. FTIR measurements were performed in order to evaluate the degree of degradation of polyamide 6 when exposed to varying intensities of radiation. The curves in the spectra were created on the basis of the median of three measurements for each test sample. The presence of chemical groups was determined via infrared spectroscopy. A comparison of IR spectra demonstrated slight material degradation, represented by the 1736 cm^−1^ belt, which is typical for carbonyl groups. Degradation is commonly also indicated by hydroxyl groups, as shown by 3500 cm^−1^ belts, but there was no trace of these groups in this area. The vibrations at 3290, 3074, 1635, 1537, 964 and 667 cm^−1^ demonstrate the presence of amide groups. The vibrations at 2920, 2852, 1463, 1352 and 1259 cm^−1^ demonstrate the presence of –CH2 groups, also indicating the presence of aliphatic chains. The intensity of these belts decreases with increasing radiation dose, which indicates decreased mobility of the –CH2 groups. The belt located in the 667 cm^−1^ area is a vibration of amide in crystalline phase α and β. Crystalline phase α is characterised by vibration with a wave number of 1201 cm^−1^, which can be attributed to the deformation vibration with a fan-like shape of –CH2. The branch of this belt increased in the irradiated test samples. The belt with the wave number 1697 cm^−1^, which is connected with CLA TAIC, was lacking in both test samples. This indicates that the addition was most likely consumed in reactions connected with PA 6 irradiation.

Table 4 shows the values of absorbance at wave number 1736 cm^−1^, which is common for carbonyl groups, and represents the degradation of PA 6. The absorbance marked in green is the lowest with a value of 0.00613, which corresponds with the results obtained for micro-mechanical properties, where sample C irradiated by 132 kGy showed the highest values of indentation hardness and elastic modulus. At the same time, the TMA results indicate that this material has the highest degree of cross-linking. The absorbance value marked in red (0.01653) belongs to test sample B irradiated by 99 kGy. This test sample showed the highest degradation.

## 4. Conclusions

In this study, the effect of CLA concentration on the indentation hardness, modulus and creep of irradiated PA 6 (66, 99 and 132 kGy) was studied. of the occurrence of cross-linking was evidenced by the thermal deformation and expansion values obtained by TMA analysis. Proof of degradation was further obtained by FTIR spectroscopy.

The micro-mechanical property results indicate that the best combinations of radiation and CLA were those in sample C irradiated by 132 kGy and in sample D irradiated by 66 kGy. The combinations resulted in the highest values of indentation hardness and indentation modulus. On the other hand, the lowest values of indentation creep were found in samples with the same TAIC concentration irradiated by 66 kGy. On the basis of the TMA measurements, it was found that the highest cross-linking of PA 6 was reached in sample C irradiated by 132 kGy. From an economic perspective, it is more advantageous to use a lower radiation intensity (e.g., sample D irradiated by 66 kGy), thus achieving high mechanical properties, but also economic relief due to fewer passes under the electron accelerator being required.

FTIR spectroscopy showed a slight degradation of the test samples, indicated by presence of carbonyl groups in vibration belt 1736 cm^−1^. The absence of the belt with the wave number 1697 cm^−1^, indicating the presence of TAIC, shows that the added CLA was consumed during the irradiation process.

Since CLA is crucial for the irradiation process, its absence means that the polymer is degrading upon irradiation. It is important to adopt correct content of this agent. It is also important to choose the correct dose of radiation.

## Figures and Tables

**Table 1:** Designation of test samples.

Sample	CLA Concentration [%]	Radiation Dose [kGy]
A	0	-
B	2	66, 99, 132
C	4	66, 99, 132
D	6	66, 99, 132

**Table 2:** Technological parameters of the injected samples.

Injection Parameter	Unit	Value
Pressure	MPa	80
Velocity	mm/s	50
Length of a dose	mm	26
Temperature under the hopper	°C	70
Cooling time	s	20
Holding pressure	MPa	68
Holding pressure duration	s	10
Heat zone settings		
Zone n. 1	°C	220
Zone n. 2	°C	230
Zone n. 3	°C	245
Zone n. 4	°C	265

**Table 3:** Parameters of TMA measurements.

Technological Parameter	Unit	Value
Starting temperature	°C	180
Dwelling time at temperature	min	10
Load	N	1
Speed of heating	K/min	1
Final temperature	°C	250

**Table 4:** Values of absorbance at wavenumber 1736 cm^−1^.

Specimen	Absorbance
A, 0 kGy	0.00979
B, 66 kGy	0.01230
B, 99 kGy	0.01653
B, 132 kGy	0.01448
C, 66 kGy	0.01219
C, 99 kGy	0.01119
C, 132 kGy	0.00613
D, 66 kGy	0.00988
D, 99 kGy	0.01225
D, 132 kGy	0.00848

## Data Availability

The data presented in this study are available on request from the corresponding author.
